# Simulation-Based Designing of Suitable Stimulation Factors for Presenting Two Phosphenes Simultaneously to Lower Side of Field of View

**DOI:** 10.3390/bioengineering9120752

**Published:** 2022-12-02

**Authors:** Manami Kanamaru, Phan Xuan Tan, Eiji Kamioka

**Affiliations:** Shibaura Institute of Technology, Graduate School of Engineering and Science, Tokyo 135-8548, Japan

**Keywords:** visually impaired people, phosphene, walking support, ocular surface, lower field of view, FEM

## Abstract

Using a phosphene has been discussed as a means of informing the visually impaired of the position of an obstacle. Obstacles underfoot have a risk, so it is necessary to inform the visually impaired. A previous study clarified a method of presenting phosphene in three directions in the lower vision; however, the simultaneous presentation of these phosphenes has not been discussed. Another study discussing the effect of electrical interference when stimulating the eyeball with multiple electrodes indicated that it is important to select appropriate stimulation factors to avoid this effect. However, when the stimulation electrodes are arranged remarkably close, there is a high possibility that the stimulus factor presented in the previous study will not apply. In this study, a method for simultaneously presenting phosphenes in the lower vision is presented. The electrode arrangements reported in the previous study to present phosphene in the lower field of vision are used, and the difficulty in the simultaneous presentation of multiple phosphenes in the lower vision is the focus. In this paper, the method of designing the stimulation factors is discussed numerically when the electrodes are arranged remarkably close. As a result, it is shown that stimulation factors different from the previous research were appropriate depending on the distance between the electrodes.

## 1. Introduction

There are at least 2.2 billion visually impaired people globally [1], and many visually impaired people use a white cane for walking. The white cane has the advantage of recognizing obstacles at the feet as a high priority; however, training is necessary for safe walking. Moreover, there is a disadvantage in that both hands cannot be used in an emergency. Therefore, studies have been conducted on a walking support device that can be used hands-free and is conscious of obstacle recognition at the feet. For example, there is a system in which sensors are attached to shoes [2] or sunglasses [3] to notify individuals of the location of obstacles by sound or vibration. However, there is a risk that visually impaired people cannot perceive the outside world if other senses, such as hearing, are hindered.

Therefore, this study focuses on the methodology of providing visual information to the lower side of the field of view to provide walking support that allows the visually impaired to recognize obstacles around their feet without hindering their senses. There is a phenomenon called phosphene, in which light flashes can be seen by electrically stimulating the brain and eyeballs [4]. This phenomenon can be recognized even by a visually impaired person by stimulating the undamaged part. Therefore, phosphene makes it possible to notify the visually impaired person of the obstacle’s position without hindering other senses.

Various noninvasive methods have been studied for eye stimulation methods. tACS (transcranial alternating current stimulation) [5], which places electrodes on the skin, TES (transcorneal electrical stimulation) [6], which places contact-lens-shaped electrodes on the cornea of the eye, and TpES (transpalpebral electrical stimulation) [7,8], which places electrodes on the eyelids, have been reported. It has also been suggested that stimulating the eyeball may lead to the treatment of retinitis pigmentosa and optic neuropathy [8,9]. Therefore, phosphene can be presented without damaging the tissue inside the eyeball by considering the stimulation value. In this study, phosphene should be presented in multiple directions to show the obstacle positions via the phosphene-based walking support for the visually impaired. However, there is a report that the presentation position of the phosphene was presented widely in the center of the visual field when using TES as a stimulation method [10]. On the other hand, the presentation position of phosphene can be changed by adjusting the electrode arrangement around the eyeball when tACS is used [4]. Several studies have reported that phosphene can be presented around the electrodes when electrodes are placed around the eyeball [11]. Therefore, in this study, the method of presenting a phosphene at the intended position using tACS is the primary focus. Since the position of an obstacle is not limited to one direction, we considered presenting phosphenes in two directions.

In our previous research, the electrode arrangement for presenting phosphenes in the right, center, and left directions of the lower side of the field of view was clarified [12]; however, presentation in two directions simultaneously was not considered. Another previous study argued that considering electrical interference is necessary when stimulating the eyeballs using two pairs of electrodes. As a coping method for electrical interference, anti-phase stimulation was effective when electrodes were stimulating both eyeballs, and the same phase was effective when stimulating one eyeball with multiple electrodes [13]. However, the previous study focused on the phosphene presentation and divided the field of view into three (right, center, left), and was intended for cases where locality was not required, as compared to this study. Moreover, the verification results are discussed based on the electric field value at arbitrary coordinates of the ocular surface, and the effectiveness of anti-phase stimulation is not discussed by focusing on the electric field value of the entire ocular surface.

Therefore, in this study, the stimulus factors (electrode arrangement, electric current, and stimulus phase) are clarified when the phosphene is presented in two directions simultaneously, including the three directions in the lower side of the field of view, and we numerically evaluate the local stimulus accuracy. By clarifying the method of presenting multiple phosphenes in the three directions of the lower visual field, visually impaired people can avoid obstacles by presenting phosphenes in the corresponding directions. Since it is possible to present the positions of multiple obstacles at their feet simultaneously, it can be seen that the safety of the visually impaired will be improved. This paper contributes to revealing a method that enables using multiple electrodes in a smaller area than in previous studies. Moreover, numerical evaluation of the locality of the stimulus position on the ocular surface enables more accurate evaluation.

The rest of the paper is organized as follows: Section 2 mentions the related work and background knowledge, and the hypothesis for stimulus factors for simulation is presented in Section 3. Section 4 describes the simulation conditions and evaluation results. Section 5 discusses the results, and the conclusions of this study are shown in Section 6.

## 2. Background Knowledge and Related Works

This section describes the knowledge of the anatomical eyeball and related studies on phosphene presentation via stimulation near the eye with an alternating current. Additionally, related studies reporting the electrical stimulation factors for phosphene presentation in specific conditions are also described.

### 2.1. Phosphene Observation and Stimulation Methods

**Figure 1:**
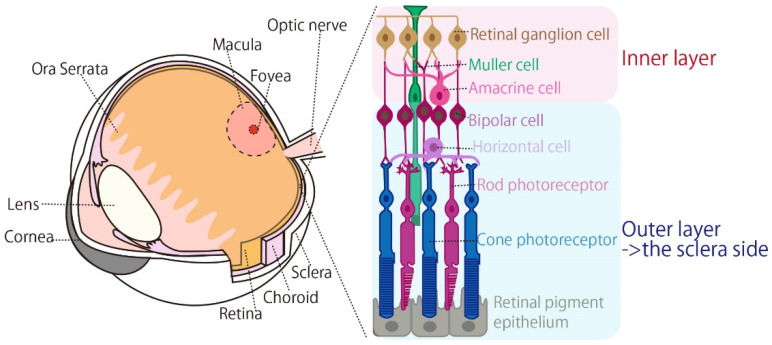
Anatomic information inside the human eyeball [8,14].

**Figure 2:**
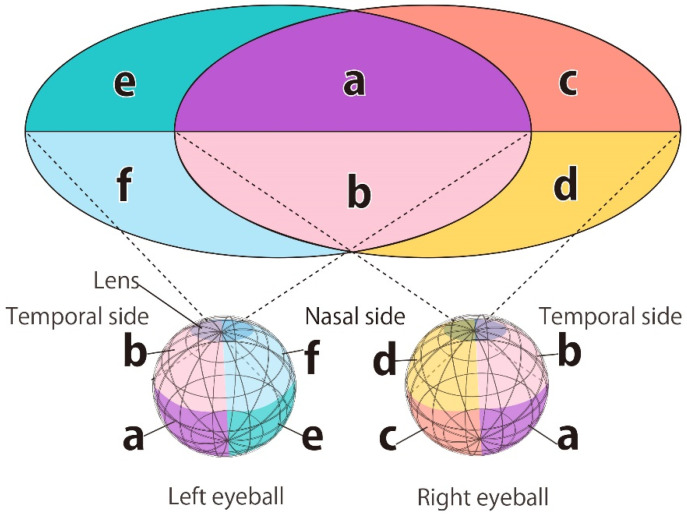
The relation between a human’s visual field and the retinal area in the eyeballs.

As shown in Figure 1, the structure of the eyeball is covered by the cornea on the skin side and the sclera on the part of the eyeball protected by the bone [8,14]. The optic nerve, fovea, and macula tissue are at the opposite pole of the cornea. The fovea and macula receive central visual information, and the area is densely populated with photoreceptors. Although there are also photoreceptors in other areas, they are not distributed from the boundary, which is called the ora serrata [4]. Photoreceptors are placed on the sclera side, which is the outer layer of the retina, and ganglion cells are distributed in the retina facing the inner side of the eyeball. Since visual information reaches the retina through the lens, it is processed by the retina on the opposite side of the actual positional information [14]. Figure 2 shows the relationship between the retinal and visual field areas. The temporal retina of both eyes at the area marked with “a” and “b” controls the central visual field. The nasal retina at the area marked with “c”, “d”, “e”, and “f” controls the peripheral visual field. For example, as shown in Figure 2, the right eye’s retina at the position marked “a” controls the upper part of the central visual field, and the area marked with “c” controls the upper right part of the visual field. In other words, phosphenes are observed on the upper right side of the visual field if the retina in the region denoted as “c” is correctly stimulated.

There have been many discussions about the stimulus position of the eyeball when a phosphene occurs. tACS, which is an eye stimulation method focused on in this study, is also used for brain stimulation [15]. Simulations reported in a related study have shown that an eyeball-originated phosphene is observed during brain stimulation and the current directly stimulates photoreceptors from the sclera side [15]. However, in another related study that observed the reaction inside the retina when the cornea was stimulated, it was reported that photoreceptors did not respond to electrical stimulation from the corneal side, and only ganglion and amacrine cells responded [16,17].

This retinal reaction contrasts the related studies [15] that show the photoreceptors can be directly stimulated by tACS. Moreover, in our previous research [4], when an electrode was placed on the upper temporal side of the right eyeball, phosphenes were presented in the purple-colored area in Figure 2 only when the retinal area “a” in Figure 2 was exposed to the skin side due to eye movement. When no eye movement was performed, it was estimated that the area “c” opposite the electrode was stimulated, and the phosphene was observed on the upper right side of the visual field. Phosphenes have been observed around electrodes placed on the face in other related studies [11,18]. In addition, it has been found that phosphenes were observed in the central visual field located opposite the electrodes when stimulated by TES [10]. Therefore, in this study, it is assumed that the presentation position of the phosphene can be estimated from the electric field value on the cornea when the eyeball does not move, and the simulation based on this is performed.

### 2.2. Phosphene Observation and Stimulation Methods

Research has been carried out on methods for controlling the presentation position of the phosphene. Several studies have reported that the position of the phosphene changes depending on the position of the electrode [4,11,18]. These follow the “hypothesis that the retina is stimulated opposite the stimulated corneal area” referred to in 2.1.

When presenting phosphenes in two directions of the visual field, multiple stimulations of limited regions of the ocular surface are required. However, since the ocular surface is a small area, if stimulated simultaneously, the alternating currents generated from the respective electrodes may interfere electrically with each other, resulting in unintended stimulation. Therefore, in the previous research [13], electric field stimulation was performed considering the electrode arrangements, stimulation value, and phase during stimulation. The verification was carried out in two stages. First, the possibility of electrical interference in the 15 patterns of electrode arrangement used in the experiment to actually present phosphenes was shown. After that, the previous study suggested that it is effective to use opposite phases when stimulating the left and right eyeballs simultaneously using an electrode arrangement for presenting phosphenes in three directions, and the same phase is appropriate when the same eyeball is stimulated by multiple electrode arrangements simultaneously. Since phosphenes tend to be observed around the electrodes [4,11,18], essentially, one electrode was installed near the corneal region intended to be stimulated and the other was installed in consideration of the unevenness of the face. The previous study indicated that the influence of electrical interference due to electrical stimulation was weakened by performing anti-phase stimulation, making it possible to stimulate only the intended area. However, in this previous study, the electrical field value on the specific coordinate of the line along the upper eyelid was observed as an evaluation method of the stimulated area on the cornea. Since the electrodes were placed on the forehead and cheeks, it is expected that the area around the observation point was stimulated; however, it cannot be determined whether the cornea as a whole was locally stimulated. Therefore, when local stimulation is applied to multiple smaller areas, it is necessary to confirm whether the anti-phase is effective when stimulating the left and right eyeballs and whether stimulating the same phase is suitable for stimulating one eyeball by multiple electrode arrangements.

Electrode arrangement for presenting phosphenes only in the lower side of the field of view has also been discussed in a previous study [12]. Based on the fact that phosphenes were observed around the electrodes, the electrodes were placed around the lower eyeball in the previous study. In order to evaluate whether the cornea on the lower eyeball could be locally stimulated, the electric field value on the surface of the eyeball was expressed as a color difference. In addition, how the electric field value over the whole ocular surface changed was discussed by converting the eyeball, which is a sphere, into a 2D image. The electrode arrangement that can stimulate the intended lower eyeball region was clarified in the previous study [12]. However, with this evaluation method, it is difficult to evaluate the local stimulation for each electrode arrangement numerically, and the current evaluation method cannot indicate the position of the ocular surface where the largest electric field value was recorded. In that case, when assuming that the phosphene is actually presented to a visually impaired person, it is difficult to estimate how much the presentation position of the phosphene can be adjusted by fine-tuning the electrode arrangement. Therefore, a method to numerically evaluate the locality of the stimulus region on the ocular surface is needed.

## 3. Method for Designing Electrode Arrangements to Induce Phosphenes in the Lower Side of the Field of View

Section 2.2 mentioned the considerations when presenting multiple phosphenes and a previous study presenting phosphenes in the lower side of the field of view [12]. The reported method of presenting multiple phosphenes simultaneously targets three directions of the visual field, and the objective of the evaluation is only minimal coordinates in the eyeball in this study. In addition, when the phosphenes are simultaneously presented in three directions in the lower side of the field of view, it is necessary to consider the effects of electrical interference to stimulate a smaller area than in the previous study [13]. This section presents hypotheses about stimulus factors, including electrode arrangement, when presenting multiple phosphenes in the lower side of the field of view.

**Figure 3:**
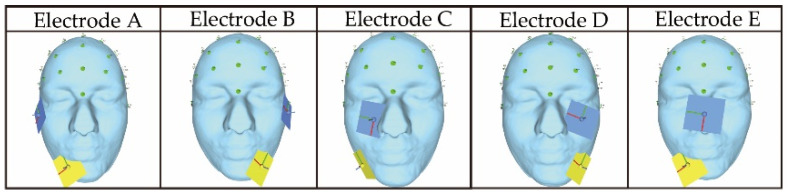
Electrode arrangements were used in the previous study for stimulating the lower human eyeball; the figures were produced by the authors using SimNIBS (03/11/2022).

Figure 3 shows the electrode arrangements used in the previous study [12] to present phosphenes in the lower side of the field of view. Using those electrode arrangements makes it possible to stimulate five areas on the lower side of both eyeballs: Temporal, central, and nasal eyeballs. In presenting a plurality of phosphenes, when stimulating both eyeballs by a combination of the electrodes shown in Figure 3 (Electrode A and Electrode B, Electrode A and Electrode D, and Electrode B and Electrode C), two electrodes are arranged on each cheek. Since the electrodes are not in close contact with each other, it is possible to stimulate the intended area via anti-phase stimulation without changing the electrode arrangement from the previous study’s arrangement. This is also inferred from the verification results of previous research [13] on phosphene presentation in two directions. However, when stimulating the same eyeball with two sets of electrodes (Electrode A and Electrode C, Electrode B and Electrode D, Electrode A and Electrode E, and Electrode B and Electrode E), it is considered that the effects of electrical interference would appear at a higher possibility because the distance between the electrodes is close. Moreover, since Electrode A and Electrode E include electrodes with common coordinates, it is necessary to arrange the yellow electrode of Electrode E on the opposite cheek. As mentioned in this section, there are four situations where the same eyeball is stimulated with multiple electrodes. There is a possibility that the same phase is suitable when stimulating the same eyeball with multiple electrodes, as mentioned in the previous study [12]. However, there would be cases where the same phase is unsuitable and the opposite phase is suitable because the stimulation condition is different from the previous study. The difference in the stimulation condition between this study and the previous study is that we are stimulating a limited area compared with the previous study in which the same phase was suitable for stimulation with multiple electrodes in the same eyeball. Therefore, in Electrode A and Electrode C and Electrode B and Electrode D, where the distance between the electrodes is the shortest, it is presumed that the effect of electrical interference outside the stimulation area will increase by performing stimulation in the same phase.

As for the current value, 1 mA is suitable as a reference because 1 mA was adopted in several previous studies [4,11,18]. Verification results have reported that it is difficult to stimulate the area on the nasal side of the eyeball, so it is necessary to consider increasing the current value. Based on the above, hypotheses were made, as shown in Table 1, regarding the method of presenting multiple phosphenes in the lower side of the field of view. In this study, simulations based on the hypotheses shown in Table 1 are performed, and selected stimulation parameters’ local stimulation is evaluated.

## 4. Evaluation

In this study, based on the results of previous studies [4] shown in 2.1, it is considered that the stimulated position on the cornea of the eyeball is related to the presentation position of phosphenes. Therefore, focusing on the value of the electric field on the eyeball cornea, we can clarify how well this study’s stimulation method can locally stimulate the intended area. This section describes the simulation method based on the hypothesis shown in Section 3, graphically showing the electric field value on the cornea for evaluation, and finally, the verification results under these simulation conditions.

### 4.1. Stimulation Factors for Simulation

In this subsection, the method of analyzing which part of the body is stimulated during electrical stimulation is outlined. After that, the stimulus factors used during the simulation in this evaluation are shown.

When considering a simulation method, the important points are the type of stimulation method and how complex tissue is targeted. For example, in this study, the target object is the ocular surface, and the stimulation value is approximately 1 mA and 10 Hz alternating current, considering previous research [4]. The reason for focusing on the ocular surface is described in 2.1. In addition, 1 mA is a value considered to have no significant side effects when stimulated with tACS in the human body [19,20], and 10 Hz is the frequency band in which intraocular flashes can be observed [4].

Typical electric field simulation methods include FDTD (Finite difference time domain method) [21] and FEM (Finite Element Method) [22]. Table 2 shows the pros and cons of FDTD and FEM. In FEM, the object is divided into small triangles or triangular pyramids called elements. In FDTD, the object is divided into a small cube, and it is characterized by the ability to analyze time-dependent electromagnetic problems. However, there is a problem in that it takes too much time to analyze the electrical stimulation in a low-frequency band [23]. In this study, a 10 Hz alternating current is considered for the stimulation current to the eyeball based on previous research [4]. Hence, using FDTD, which focuses on the frequency band of 100 Hz or higher [24], is inappropriate. In addition, since FDTD uses a cube to divide the area, there is a problem regarding the error due to staircase approximation in the case of analysis of a complicated object with many curves. On the other hand, FEM divides the object into triangular or pyramidal elements, so even complex shapes of an object can be handled. Although the high computational cost is a disadvantage of FEM, it has been used in previous research to clarify the stimulation position of the eyeball because it can be analyzed even in the low-frequency band.

SimNIBS is human body electric field simulation software using FEM [25,26,27,28,29]. SimNIBS is a free simulation software for non-invasive stimulation and has been used in previous studies [12,13]. The human body to be simulated is divided into a surface and a volume, with the surface being divided into triangular elements and the volume into triangular pyramids for simulation, making it possible to handle complex human bodies. Users of SimNIBS themselves can prepare a specific head mesh model and perform simulations. On the other hand, SimNIBS provides a head mesh model, Ernie, a healthy male, as a sample model. Ernie’s face uses the average data of five subjects for privacy protection. Written informed consent was given to use the MR (magnetic resonance) data, and the data were anonymized. Radiologists have confirmed this treatment. Details of Ernie’s dataset are shown in Table 3. SimNIBS can simulate two types of stimulation, as shown in Table 3. Although tDCS uses direct current, it is a similar stimulation method to tACS. In this study, the target is stimulation at 10 Hz; however, since it is in a low-frequency band, the developer of the simulation software mentioned that it is possible to use the simulation results of tDCS.

The Neumann boundary condition is used for boundary conditions in the numerical calculation of SimNIBS. Dirichlet boundary conditions are used only in the region of the electrodes in tDCS. In SimNIBS, the electrical conductivity of each tissue is given as an initial value, but the electrical conductivity changes depending on the stimulation frequency. Therefore, any electrical conductivity can be set by the software user. The values, used in this study are shown in Table 4.

In addition, when simulating the electric field value on the cornea of the eyeball, the default electric field value of SimNIBS is used in this study. This electric field value is the magnitude of the electric field vector of each element and has a positive value. As mentioned in 2.2, a previous study [applied] clarified the electrode arrangement that presents phosphenes in three directions on the lower side of the field of view. In this study, multiple electrode arrangements are used simultaneously, so it is presumed that stimulating the target area using the electrode arrangement of the previous study is difficult.

**Figure 4:**
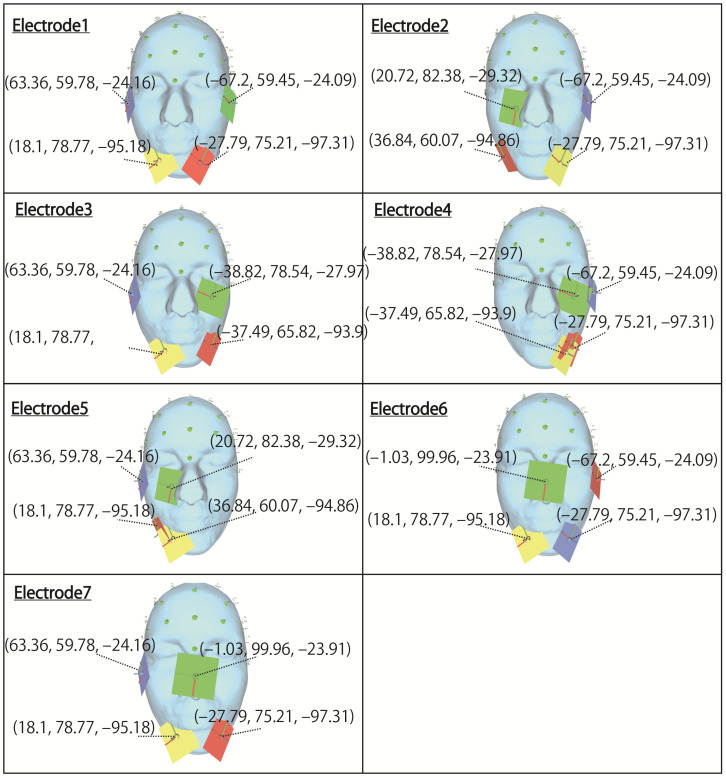
Electrode arrangements to stimulate the lower eyeballs with multi electrodes.

However, in this evaluation, to confirm whether the stimulation factors hypothesized in Table 1 are adequate, simulations are performed using the electrode arrangements shown in Figure 4, referring to the electrode arrangements of the previous study. Electrodes 1, 2, and 3, which stimulate the right and left eyeball, respectively, use the same coordinates as the electrode arrangement used in the previous study. Electrodes 4, 5, 6, and 7, in which a plurality of electrodes are arranged near the same eyeball, likely need to be changed because the electrodes are close to each other. In particular, for electrode 7, which is considered to be impossible to arrange because the coordinates of the electrodes are in the same position, stimulation can be performed by moving the electrode to the opposite cheek, as shown in Figure 4. Although electrodes 4 and 5 are close to each other, they do not overlap completely, so the electrode arrangement in the previous study is used. The coordinates in the table indicate the x, y, and z coordinates from the left, with the origin being the center of the head mesh model in the figure. Regarding the stimulus current, 1 mA is essentially used according to previous studies [4,12,13]. As mentioned in 2.2, it may be necessary to increase the current value depending on the stimulation site. The previous study showed simulation results at 1.5 mA to stimulate the nasal eyes, so it is necessary to adjust this current value as a reference. Regarding the phase, as mentioned in Section 3, the anti-phase is not optimal for all electrode arrangements, and it is necessary to consider the possibility that there are electrode arrangements for which the same phase is suitable. Therefore, same-phase and anti-phase stimulation is performed for all electrode arrangements. This simulation procedure makes it possible to verify which stimulation factor is more suitable. The electrode design in the simulation reproduces the electrode of “foc.us”, which is a stimulator used in previous research [4] and has a size of 42 mm × 42 mm [32]. By making the part facing the skin a gel pad, it is possible to make it closer to the actual electrode of “foc.us”.

### 4.2. Evaluation Methodology

**Figure 5:**
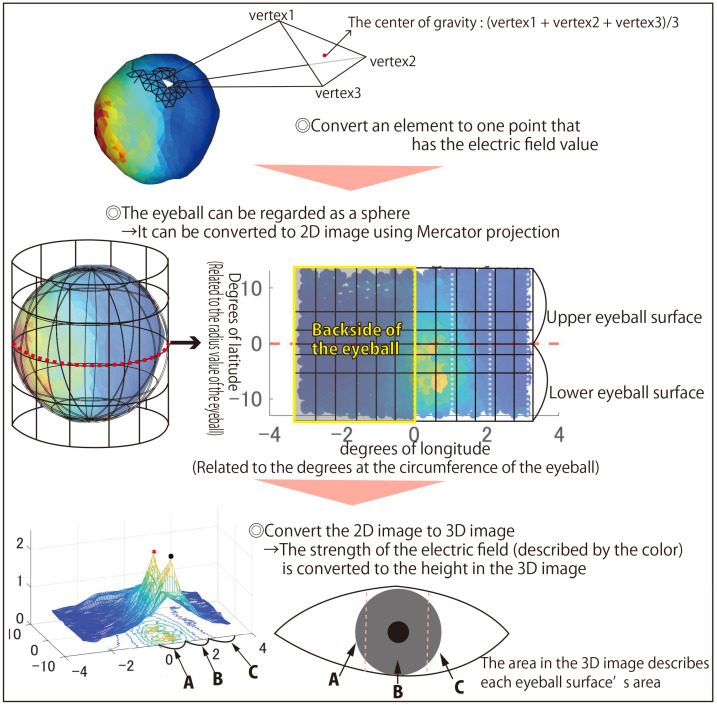
Evaluation methodology of the electric field simulation for showing the locality stimulation.

In this subsection, a method for numerically evaluating the simulation results is described, which is shown in Figure 5. SimNIBS performs electric field simulation using FEM as mentioned in 4.1 and calculates it by dividing each tissue into triangles and triangular pyramids. The stimulation position of the eyeball’s cornea is necessary for this research, so if the electric field value of the elements that make up the surface of the eyeball is obtained, the simulation results can be illustrated. In SimNIBS, the elements on the tissue’s surface are triangular, so the surface of the eyeball is covered with small triangles, and each triangle element stores an electric field value. When evaluating the stimulus position of a spherical eyeball, it is difficult to observe all corneal surfaces simultaneously if the eyeball has a spherical shape. Therefore, in the previous study [12], the one triangular element was replaced with one point that has one electric field value. Moreover, the points replaced by the elements on the ocular surface were converted into a plane using the conversion method of Mercator projection and evaluated the stimulus position. This made it possible to evaluate whether or not the target area was stimulated intuitively. However, it is difficult to evaluate the stimulus position numerically with this conversion technique. In this study, the electrode arrangement in which the area to be stimulated and the area not to be stimulated alternately exist in the same eyeball is also focused on, so it is necessary to be able to numerically evaluate the extent to which other areas are not affected. Therefore, in this study, as shown in Figure 5, the electric field value of the ocular surface used in the previous study was converted into a flat surface, and one more analysis of the experimental results was performed. The final step of the evaluation method is converted into a 3D graph in which the *x*-axis is the longitude when the eyeball is regarded as a sphere, the *y*-axis is the latitude, and the *z*-axis is the electric field value. When converting to a 3D graph from the scatter 2D graph, the grid mesh is interpolated between the points extracted from the elements with each electric field value. If the 3D image is converted from the right eyeball’s elements, area A, depicted in Figure 5, represents the temporal right eyeball, area B represents the central right eyeball, and area C represents the nasal right eyeball. For example, when the intended stimulus area is the lower ear side of the right eyeball, if the peak of the electric field value of the right eyeball exists in the area between 0 and 1.047 in the x-coordinate (area A in Figure 5) and 0 or less in the y-coordinate, the intended ocular surface can be stimulated correctly. Suppose the difference between the maximum electric field value in the region of the eyeball surface intended to be stimulated and the maximum electric field value in the adjacent region is large. In that case, it is determined that the adjacent region is less affected by the stimulation. In this way, it is assumed that local stimulation with the selected stimulation factors has been achieved when the stimulation has little effect on adjacent regions.

The point with the maximum electric field value within the stimulation region is marked with a black circle, and the point with the maximum electric field value outside the stimulation region is marked by a red star. The area above the eyeball of the stimulation area is identified as “upper”, and the area adjacent to the stimulation area is identified as “adjacent”.

### 4.3. Evaluation Result

**Figure 6:**
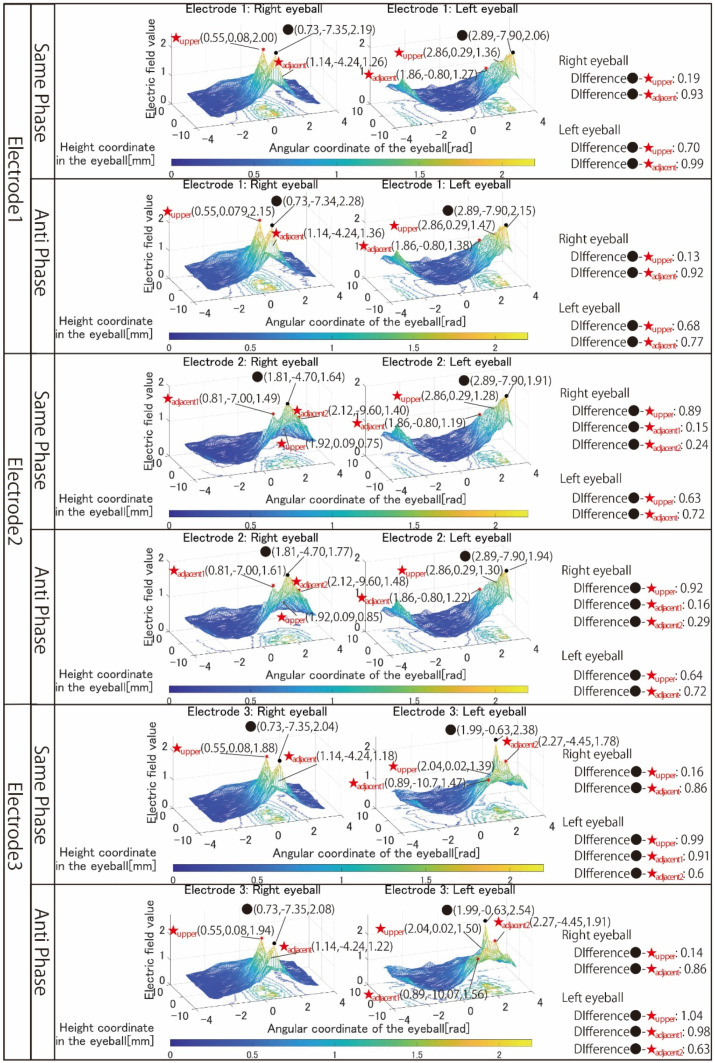
Simulation results with the electrode arrangement to stimulate both eyeballs.

**Figure 7:**
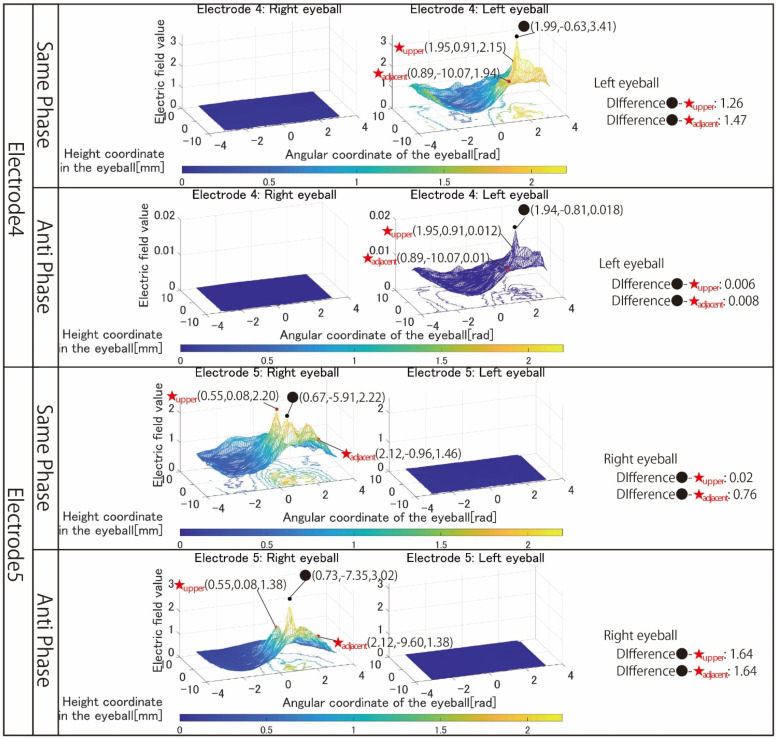
Simulation results with the electrode arrangement to stimulate the temporal and central eyeball.

**Figure 8:**
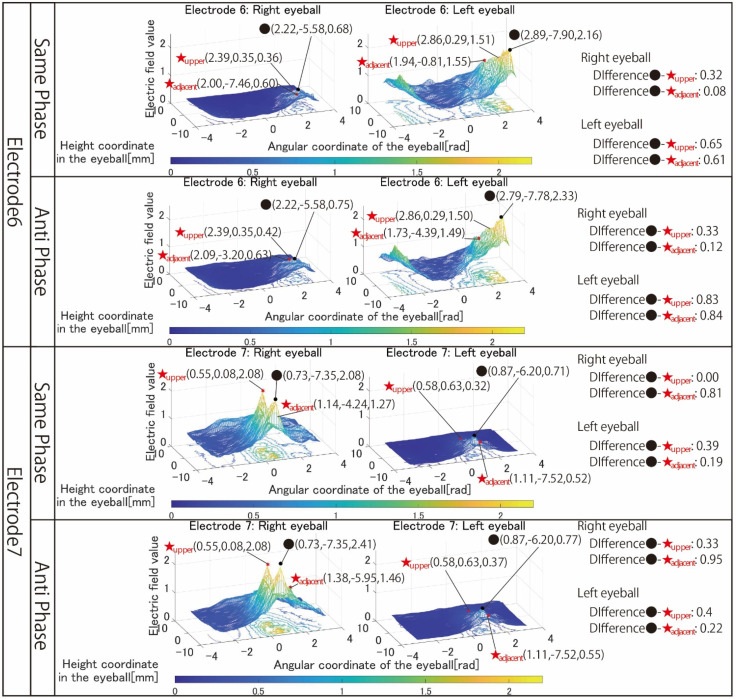
Simulation results with the electrode arrangement to stimulate the temporal and nasal eyeballs.

Figure 6 shows the simulation results with electrodes 1 to 3 that stimulated both eyeballs, respectively. Figure 7 shows the simulation results with electrodes 4 and 5 that stimulated one eyeball with two pairs of the electrode arrangement. Figure 8 shows the simulation results using electrodes 6 and 7 that stimulate the nasal side of the eyeball.

The *x*-axis of all results is the angular coordinate, the *y*-axis is the height coordinate, and the *z*-axis is the electric field value. The maximum value of the *Z*-axis is the optimum value for the simulation content, but the colors of the mesh that change depending on the electric field value are all fixed. This makes it possible to clarify the change in the electric field value due to the difference in the stimulation factors from the color of the mesh, numerical values, and shape of the 3D image. The coordinates of the point with the maximum electric field value written in the figures of the simulation result are x, y, and z from the left.

First, the results in Figure 6 are focused on. There is no noticeable change in the shape of the 3D image, whether using same-phase or anti-phase stimulation. Comparing the maximum value of the electric field value within the stimulation area and the maximum value in the adjacent stimulation area, it can be seen that anti-phase stimulation has a larger value than same-phase stimulation, but both difference values are small.

In the many cases shown in Figure 6, the difference between the maximum electric field values in the stimulation area and outside the stimulation area calculated under each simulation condition is larger during anti-phase stimulation. In other words, the rapid attenuation of the electric field value toward the outside of the stimulation area is considered to be same-phase stimulation with a large difference between the electric field values of the stimulation area and the outside of the stimulation area. Therefore, from the viewpoint of local stimulation, same-phase stimulation was more effective.

Next, the results in Figure 7 are focused on. The simulation results of electrode 4 show that the left eyeball is strongly stimulated only in same-phase stimulation. Although it appears as a waveform with anti-phase stimulation, the maximum electric field value in the stimulation area is minimal at 0.018, and the color of the mesh indicating the level of the electric field value also shows that the left eyeball cannot be stimulated. On the other hand, looking at the results of electrode 5, it can be seen that the right eyeball can be stimulated in either phase. Electrode 5 is an electrode arrangement that stimulates the temporal and center of the right eyeball, but areas other than the stimulation area are also widely stimulated during same-phase stimulation. This is also evident from the difference between the maxima within the stimulus area and outside the stimulation area. During same-phase stimulation, the difference in the upper eyeball area was 0.02, and for the adjacent nasal right eyeball, the difference was 0.76. The difference in the area on the nasal side of the right eyeball is 1.64 during anti-phase stimulation, and it can be seen that the electric field value sharply decreases toward the outside of the stimulation area during anti-phase stimulation. Therefore, it can be seen that anti-phase stimulation is more appropriate for an electrode arrangement such as electrode 5 that stimulates a close region of the ocular surface. As can be seen from the fact that the maximum value of the stimulation area was 3.02 during anti-phase stimulation with electrode 5, it is presumed that the difficulty in stimulating the left eyeball with electrode 4 was due to the electrode arrangement. Finally, the results in Figure 8 are focused on. It can be seen that both electrodes 6 and 7 have difficulty stimulating the nasal eyeballs. For example, during same-phase stimulation of electrode 6, the maximum value in the region on the temporal side of the left eyeball is 2.16, whereas the electric field value on the nasal side of the left eyeball drops significantly, and the maximum value in the nasal right eyeball is 0.68. There is a high possibility that the same degree of phosphenes cannot be recognized compared to the stimulus value on the temporal side of the left eyeball. Therefore, as mentioned in Section 3, it is necessary to increase the current value of the electrode that stimulates the nasal eyeballs. In addition, as can be seen in electrode 7, the maximum value of the electric field value in the stimulation region coincides with the maximum value in the upper side of the stimulation region during same-phase stimulation. In electrodes 1, 3, and 5, which are the other electrode arrangements for stimulating the temporal right eyeball, a peak of the electric field was observed in the region upper eyeball, although the maximum value of the stimulation region was not reached. Therefore, it is necessary to reconsider the electrode arrangement for stimulating the temporal lower right eyeball and its stimulation value.

## 5. Discussion

In the simulation results shown in Section 4, some electrode arrangements did not achieve local stimulation of the intended area. This section shows what kind of countermeasures are effective for those electrode arrangements and simulates them to discuss multiple phosphene presentation techniques in the lower side of the field of view. The conditions in which there were problems with local stimulation listed in Section 4 can be broadly classified into three categories:

- An electrode arrangement that stimulates the temporal and center of the left eyeball
- The position of the electrode near the eyeball of the electrode arrangement stimulates the temporal right eyeball.
- The current value and phase in the electrode arrangement, including the electrodes that stimulate the nasal eyeballs.

**Figure 9:**
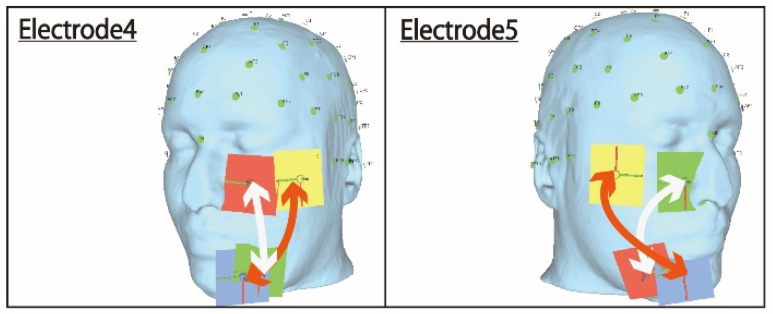
Comparison of the stimulus pathway between electrodes 4 and 5.

The first condition is discussed first. A comparison of electrode arrangements 4 and 5 shown in Figure 9 reveals that the electrodes on the lower side of the face overlap significantly in electrode 4, which failed, compared to electrode 5, which was successfully stimulated. Furthermore, the lines shown in Figure 9 are reference paths for the electric current flow between the pair of electrodes. Looking at the electric current pathway, it can be seen that the crossing points of the stimulation pathways between the two electrodes are different. Electrode 4 crossed on the lower electrode, and it was speculated that the stimulus pathways crossed at that position and that the electric field values canceled each other due to anti-phase stimulation. This can also be inferred from the fact that the ocular surface was stimulated if electrode 4 was stimulated in the same phase. Therefore, the problem can be improved by adjusting the coordinates of electrode 4 regarding the positional relationship with electrode 5.

In addition, from the simulation results shown in Figure 6, stimulation in the anti-phase during stimulation by electrode 5 was optimal because local stimulation was possible. Therefore, anti-phase stimulation is appropriate for the electrode arrangement that stimulates the temporal and central eyeball.

The condition of the electrode for stimulating the temporal right eyeball, which is the second problem, is mentioned below. At electrodes 1, 3, 5, and 7, which are electrode arrangements that stimulate the temporal right eyeball, the maximum electric field value in the stimulation area of the right eyeball does not differ significantly from the maximum value in the stimulation area’s upper eyeball. It can be seen that the difference between the maximum value on the temporal side of the right eyeball and the maximum value on the upper side of the stimulation area is not sufficient. It is also evident from the difference between the maxima in the upper region shown in Figure 6, Figure 7 and Figure 8. In other words, if the current value is simply reduced, the maximum value of the stimulated area is also assumed to be similarly reduced, and there is a possibility that the purpose of local stimulation cannot be achieved. On the other hand, since the peak in the upper stimulation region attenuates sharply toward the upper eyeball, the position of the electrode that stimulates the temporal right eyeball may be adjusted to eliminate the effect on the upper right eyeball region.

Finally, the third problem condition is mentioned, regarding the current values and phases in the electrode arrangement, including the electrodes stimulating the nasal eyeballs. Previous research has reported that stimulation of the ocular surface with electrodes that stimulate the nasal side of both eyes is more difficult than other stimulation areas [13]. In a previous study, it was said that in the case of an electrode arrangement such as electrode 6, it was possible to stimulate both regions by stimulating the electrode on the temporal left eyeball with 1 mA and the electrode on the nose side with 1.5 mA. However, looking at the simulation results of the corresponding electrode arrangement in Figure 8, for example, with electrode 6 in same-phase stimulation, the maximum value of the stimulation area on the nasal side is 0.68 and the maximum value of the stimulation area on the temporal side is 2.16, which is a significant difference. In other words, even if the nasal region is stimulated with 1.5 mA, the maximum value in the nasal region cannot be stimulated to the same extent as the maximum value in the temporal region. Therefore, by adjusting the current value of the electrode that stimulates the area on the temporal side to a smaller value, the same level’s extent of phosphenes can be observed more easily.

**Figure 10:**
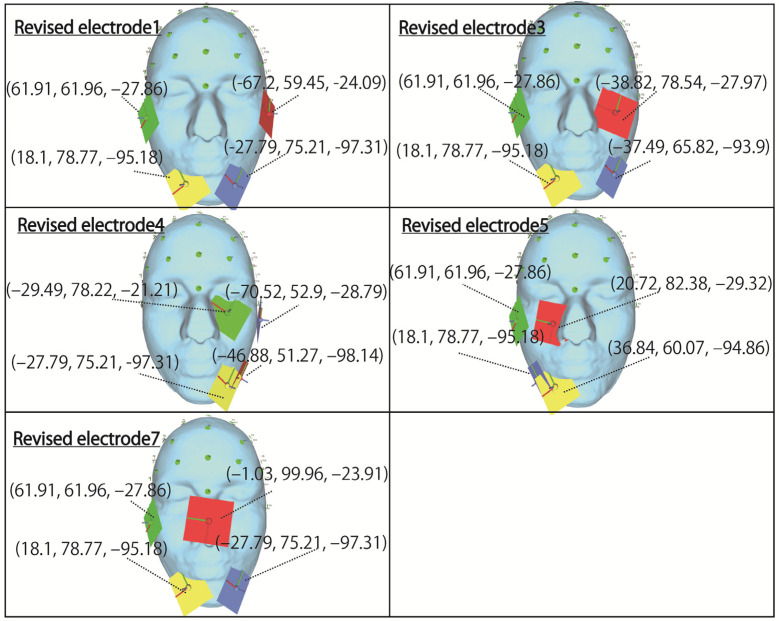
Revised electrode arrangements to improve the local stimulation.

Therefore, re-simulation is performed based on the countermeasures described above for the three conditions that had problems in the simulation results shown in Section 4. The electrode arrangement for stimulating the temporal and central left eyeball, which is the first condition, is shown in revised electrode 4 in Figure 10. For the stimulus value, we continue to use 1 mA. Moreover, when the distance between the electrodes is so close that they are in close contact, it is clear from the simulation results for electrode 5 in Figure 7 that anti-phase stimulation is appropriate, so in this section, only the anti-phase simulation results are shown.

The electrode arrangement on the temporal right eyeball, which was the second condition, is shown in Revised electrodes 1, 3, 5, and 7 in Figure 10. For Revised electrodes 1, 3, and 5, 1 mA was still used as the stimulus value, and both same-phase and anti-phase simulation results are shown.

Finally, the third condition is mentioned, regarding the stimulus value that stimulates the nasal side of the eyeball. With the aim of making the brightness of the phosphene by stimulating the temporal and nasal side of both eyes as similar as possible, the stimulation value for the temporal electrode is 0.8 mA and the stimulus value for the nasal electrode is 1.5 mA. In the case of an electrode arrangement that stimulates the nasal side, both same-phase and anti-phase simulation results are shown.

**Figure 11:**
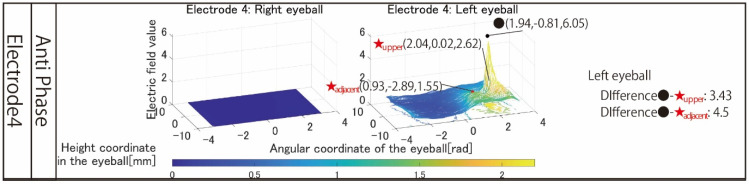
Simulation result with revised electrode arrangement to stimulate left eyeballs.

Figure 11 shows the simulation results using a revised electrode that stimulates the temporal and center of the left eyeball. Unlike the results in Figure 7, the left eyeball can be stimulated. The difference between the maximum electric field values in the stimulation area and the upper area is 3.43, and it can be seen that the electric field value rapidly attenuates toward the upper side of the eyeball. This rapid attenuation was also observed in the adjacent region, and ideal local stimulation could be realized. A point of concern is that the maximum electric field value in the stimulated region is as high as 6.05 despite stimulation at 1 mA. It is conceivable that this phenomenon may cause the perceived brightness to be different from that of phosphenes produced by other electrode arrangements. In that case, setting the stimulus value to a small value is necessary.

**Figure 12:**
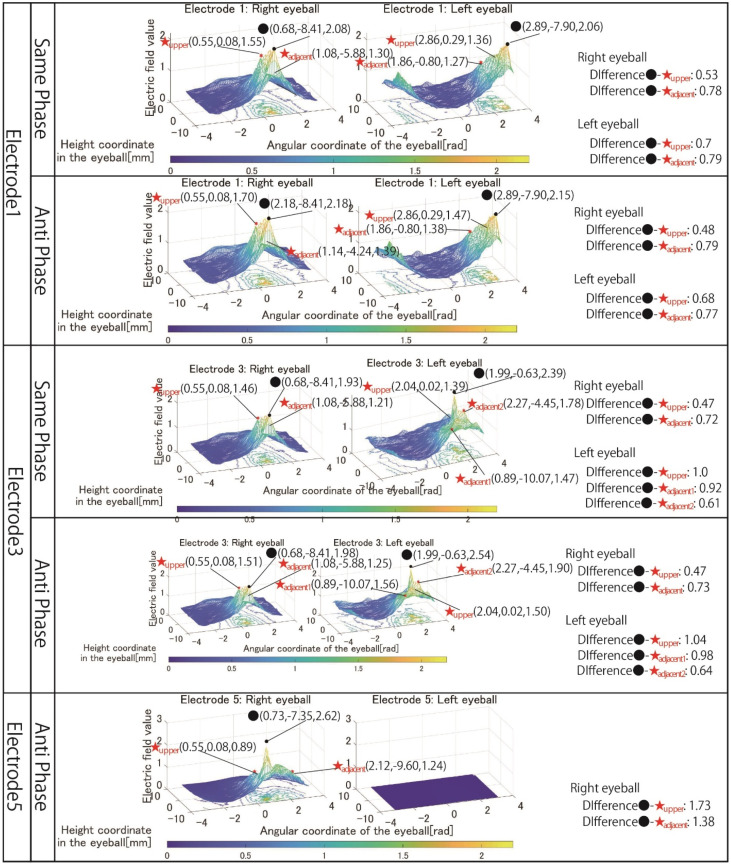
Simulation result with revised electrode arrangement to stimulate temporal right eyeball.

Figure 12 shows the simulation results for electrodes 1, 3, and 5, among the electrode arrangement, including the electrode for stimulating the temporal right eyeball, where it is considered unnecessary to change other factors, such as the stimulation value. Electrodes 1 and 3 were stimulated with 1 mA, and both same-phase and anti-phase stimulation results are shown. As for electrode 5, since the electrodes are placed in close contact with each other, the result of stimulating only in the anti-phase is shown. In any pattern of electrodes 1, 3, and 5, it can be seen that the effect of the electric field value on the region upper the right eyeball is suppressed compared to the simulation results shown in Section 4. For example, looking at the result of same-phase stimulation of electrode 1, the difference between the maximum electric field value in the stimulated region of the right eyeball and the maximum electric field value in the region upper the eyeball is 0.53. Considering that the difference in the electric field value between the stimulation area and the upper area in the results of electrode 1 shown in Figure 6 was 0.19, adjusting the electrode arrangement significantly increased the electric field value to the area above the eyeball. It can be seen that the influence of the electric field value in the upper area of the stimulation area was improved, and the local stimulation was achieved in the temporal lower-right eyeball.

**Figure 13:**
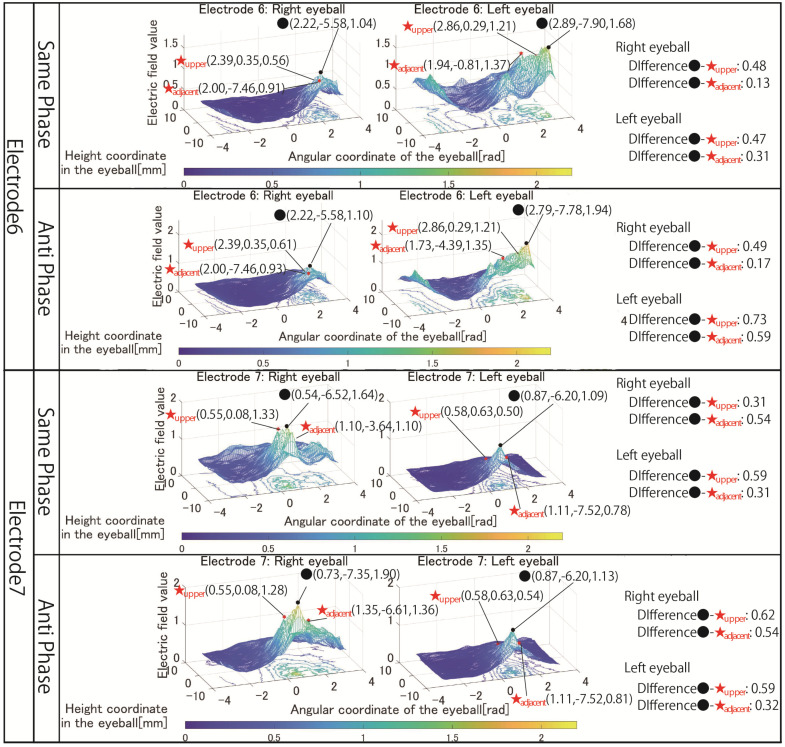
Simulation result with revised electrode arrangement to stimulate temporal nasal eyeballs.

Figure 13 shows the simulation results for electrode arrangements 6 and 7, including electrodes that stimulate the nasal side of the eyes. Compared to the simulation results shown in Figure 8, it can be seen that the area on the nasal eyeball can be reliably stimulated by adjusting the current value. In addition, this change in current value more clearly shows the difference in stimulus locality between same-phase and anti-phase stimulation. When stimulated in the anti-phase, the electric field value is high not only in the eyeball’s temporal side and nasal side regions but also in the central region, and the electric field value gradually attenuates from the temporal side to the nasal side. On the other hand, it can be seen that the mesh is wavy, avoiding the central area of the eyeball when stimulated in the same phase. Comparing the maximum values of the stimulated regions on the temporal eyeball and the nasal eyeball, for example, at the time of same-phase stimulation of electrode 6, the values are 0.68 and 2.16 in Figure 8, resulting in a difference of 1.48. On the other hand, Figure 13 shows values of 1.04 and 1.68, and the difference has been reduced to 0.64. Therefore, by setting the electrode stimulating the temporal side to 0.8 mA and the electrode stimulating the nasal side to 1.5 mA, the phenomenon that the nasal side was not significantly stimulated was improved.

## 6. Conclusions

This study clarified a method of presenting phosphenes in two directions of the lower side of the field of view to realize walking support for the visually impaired by providing visual information. As a result of verification via simulation, the following matters were clarified:

- If multiple electrodes are placed in close contact, it is necessary to consider the current pathway and adjust the electrode arrangement so that the stimulation currents do not cancel each other out. In addition, anti-phase stimulation is suitable when the electrodes are in close contact with each other.
- When one pair of electrodes is placed under the right eyeball and the other under the left eyeball, the effect of the phase difference on the stimulation area is small. Same-phase stimulation is suitable if more local stimuli are considered.
- When stimulating the nasal lower eyeball and another area simultaneously, it is necessary to increase the stimulation value of the electrode that stimulates the nose side and the other areas with a small value. In addition, when the electrodes are not arranged in close contact with each other, same-phase stimulation is suitable even when a plurality of electrodes stimulates one eyeball.

The above findings did not include the stimulation conditions presented in a previous study [12] that stimulated the eyeballs simultaneously with multiple electrodes. Clarifying those undiscovered factors when multiple electrodes stimulate the lower eyeballs was the contribution of this study. In order to stimulate small, divided regions of the ocular surface simultaneously and locally, it is necessary to design stimulation factors based on the findings shown in this study. In the future, the phosphene-based walking support system can be realized by clarifying a method for easily designing these complex stimulation factors by calculating backward from the eyeball area to be stimulated.

## Figures and Tables

**Table 1:** Hypotheses about the adequate stimulation factors to stimulate lower human eyeballs with multiple electrodes.

Stimulus Position	Electrode Arrangement	Electric Current Value	Phase
Temporal right eyeball Temporal left eyeball	Electrode A & Electrode B	1 mA & 1 mA	Anti-phase: suitable Same-phase: unsuitable
Central right eyeball Temporal left eyeball	Electrode B & Electrode C	1 mA & 1 mA	Anti-phase: suitable Same-phase: unsuitable
Temporal right eyeball Central left eyeball	Electrode A & Electrode D	1 mA & 1 mA	Anti-phase: suitable Same-phase: unsuitable
Central left eyeball Temporal left eyeball	Electrode B & Electrode D	1 mA & 1 mA	Anti-phase: suitable Same-phase: unsuitable
Temporal right eyeball Central right eyeball	Electrode A & Electrode C	1 mA & 1 mA	Anti-phase: suitable Same-phase: unsuitable
Nasal left eyeball Temporal left eyeball	Electrode B & Electrode E	1 mA & 1 mA (1 mA & 1.5 mA)	Anti-phase: unsuitable Same-phase: suitable
Temporal right eyeball Nasal right eyeball	Electrode A & Electrode E (Lower electrode of Electrode E should be moved to left side of face)	1 mA & 1 mA (1 mA & 1.5 mA)	Anti-phase: unsuitable Same-phase: suitable

**Table 2:** The characteristics of simulation methods for the electric field.

Simulation Method	Pros	Cons
FDTD	Possible to simulate a time-related problem	Time-consuming of simulation in low-frequency band Low accuracy of simulation using a complex object
FEM	Possible to simulate using complex object	High computational cost

**Table 3:** Conditions of Ernie head mesh dataset [25].

Parameters	Detail Information
voxel sizes	T1- and T2-weighted images: 1 × 1 × 1 ${\mathrm{mm}}^{3}$
voxel sizes Targeting as a stimulation method	diffusion MR images: 2 × 2 × 2 ${\mathrm{mm}}^{3}$
	TMS (transcranial magnetic stimulation) tDCS (transcranial direct current stimulation)

**Table 4:** The setup electric conductivity value obtained with 10 Hz stimulation [30,31].

Tissues	Setup Value of Conductivity (10 Hz) [S/m]
White matter	0.027656
Gray Matter	0.027512
Cerebral Spinal Fluid	2.0000
Bone	0.020028
Head skin	0.0002
Eyeball	0.41113

## Data Availability

Publicly available datasets were analyzed in this study. These data can be found here: https://simnibs.github.io/simnibs/build/html/dataset.html (accessed on 18 November 2021).
